# P2X7 Receptor Expression in Patients With Serositis Related to Systemic Lupus Erythematosus

**DOI:** 10.3389/fphar.2019.00435

**Published:** 2019-04-29

**Authors:** Federica Furini, Anna Lisa Giuliani, Mattia Erminio Parlati, Marcello Govoni, Francesco Di Virgilio, Alessandra Bortoluzzi

**Affiliations:** ^1^Section of Rheumatology, Department of Medical Sciences, University of Ferrara and Azienda Ospedaliero-Universitaria Sant’Anna di Ferrara, Cona, Italy; ^2^Department of Morphology, Surgery and Experimental Medicine, University of Ferrara, Ferrara, Italy

**Keywords:** P2X7R, Systemic Lupus Erythematosus, NLRP3 inflammasome, IL-1β, IL-6, serositis

## Abstract

**Introduction:** P2X7R is an extracellular ATP-gated receptor involved in inflammatory and autoimmune processes mainly acting through NLPR3-inflammasome activation and IL-1β release, also implicated in lymphocyte proliferation and cellular apoptosis. Several observations from animal models and patients’ studies highlight a possible link between P2X7R-NLRP3 axis and Systemic Lupus Erythematosus (SLE) pathogenesis. The P2X7R-inflammasome axis in addition to the direct production of IL-1β and IL-18, indirectly mediates the release of other cytokines implicated in the pathogenesis of SLE, such as IL-6. The aim of this study was to investigate the role of P2X7R and NLRP3-inflammasome in SLE.

**Methods:** Forty-eight SLE patients, 16 with (SLE-S) and 32 without (SLE-NS) history of serositis, and 20 healthy control (HC) subjects were enrolled. Demographic, clinical, and therapeutic data were collected. IL-1β and IL-6 plasma levels were evaluated by ELISA. Peripheral blood mononuclear cells (PBMCs) were isolated from venous blood by Ficoll gradient sedimentation and employed as follows: (1) evaluation of P2X7R and NLRP3 expression by RT-PCR; (2) determination of P2X7R activity as Benzoyl ATP (BzATP)-induced [Ca^2+^]_i_ increments using Fura-2-AM fluorescent probe; (3) isolation of monocytes/macrophages and assessment of *in vitro* IL-1β and IL-6 release following stimulation with lipopolysaccharide (LPS) and BzATP, either separately or in combination.

**Results:** Plasma IL-1β levels were unmodified in SLE respect to HC whereas IL-6 levels were higher in SLE than in HC, resulting significantly increased in SLE-S. Macrophages isolated from SLE patients released lower quantities of IL-1β after stimulation with BzATP, whereas IL-6 release was significantly augmented in SLE-NS respect to both HC and SLE-S after all types of stimulation. The [Ca^2+^]i increase following BzATP stimulation was significantly lower in PBMCs from SLE patients than in PBMCs from HC. RT-PCR showed significantly reduced P2X7R and significantly augmented NLRP3 expression in PBMCs from SLE patients.

**Conclusion:** Our data indicate reduced P2X7R expression and function in SLE patients compared with HC and, conversely, increased IL-6 signaling. The possible consequences of reduced P2X7R, mainly on cytokines network deregulation and lymphocyte proliferation, will be further investigated as well as the role of IL-6 as a possible therapeutic target especially in lupus serositis.

## Introduction

With the first reports of Burnstock in 1970s, adenosine triphosphate (ATP) has passed from a simple molecule devoted to energy reserve, to a relevant extracellular signaling molecule able to mediate numerous physiological and pathological processes (Burnstock, 2006).

Under physiological conditions ATP is poorly present in the extracellular space reaching concentrations in the order of nanomolar (nM). On the contrary, in pathological conditions ATP can behave as a danger associated molecular pattern (DAMP) being released from damaged or dying cells or from intact cells following either stimulation or mechanical or oxidative stress (Pellegatti et al., 2005; Burnstock, 2006; Giuliani et al., 2018).

P2X7 receptor (P2X7R) is the most investigated and well defined receptor for extracellular ATP.

P2X7R forms a homo-trimer ion channel allowing the efflux of K^+^ and influx of Na^+^ and Ca^2+^ after low ATP activation. After prolonged stimulation by high ATP, the receptor forms a pore that allows the passage of hydrophilic solutes of molecular weight up to 900 Da (Virginio et al., 1999). P2X7R expressed in all immune cells including monocytes/macrophages, T and B lymphocytes, dendritic cells (DCs), mast cells and natural killer cells. Its role in NLRP3 inflammasome activation and IL-1β release, is crucial for inflammatory responses (Di Virgilio et al., 2017). P2X7R activation by extracellular ATP provokes the assembly of NLRP3 inflammasome which determines the activation of procaspase-1 to caspase-1, which in turn mediates the cleavage of pro-IL-1β and pro-IL-18 to their active forms (IL-1β and IL-18) subsequently released into the extracellular space (Piccini et al., 2008). Given the powerful IL-1β-mediated inflammatory response involved in many pathological processes (Dinarello, 2009), production and release of this cytokine is strictly controlled first of all by regulation of the inflammasome. The NLRP3 inflammasome is able to directly determine the production of IL-1β and IL-18. With the activation of P2X7R, however, there is also the release of other inflammatory cytokines such as IL-1α, TNF-α, and IL-6, independently of the inflammasome (Gicquel et al., 2015).

In addition, P2X7R is involved in regulation of cell growth and proliferation. In particular, through Ca^2+^ influx P2X7R is able to activate NFAT (nuclear factor of activated T cell) that promotes transcription of the IL-2 gene and consequent T lymphocytes proliferation (Yip et al., 2009). NFAT mediates activation of two relevant transcription factors: the nuclear factor kappa B (NF-κB), involved in both pro-inflammatory cytokines (IL-1β, IL-18, IL-6, IL-8, and TNF-α) production (Liu et al., 2011)and apoptosis, and the hypoxia inducible factor 1α (HIF-1α) released during hypoxia, also activated during neoplasms allowing transcription of multiple genes involved in apoptosis resistance, inflammation, angiogenesis, tumor invasiveness and metastasis (Tafani et al., 2014).

P2X7R therefore, displays a dual role depending on both the degree of activation by ATP (in terms of concentration and duration of stimulation), and the expression of the receptor itself that can vary between different cells. The level of P2X7R expression and the cell type where it is expressed may induce a prevalent pro-proliferative or pro-apoptotic activity (Adinolfi et al., 2012). Thanks to all these characteristics, P2X7R might become an important therapeutic target in inflammatory and neoplastic diseases.

Systemic Lupus Erythematosus (SLE) is traditionally considered the prototype of autoimmune diseases in which adaptive immunity is the main driver characterized by an aberrant activation of auto-reactive B-lymphocytes and consequent production of autoantibodies, and immuno-complexes formation. Recent evidence emphasize a possible role of innate immunity, and in particular of the purinergic system, in the pathogenesis of SLE (Bortoluzzi et al., 2016; Di Virgilio and Giuliani, 2016). Several mouse models (NZB/WF1, MLR/lpr, BXSB/Yaa, and pristane-induced) and some data in humans provided demonstration of the role of innate immunity and especially of P2X7R-NLRP3 inflammasome, in SLE pathogenesis (Turner et al., 2006; Zhao et al., 2013). Innate immunity can use processes of programmed cell death as a form of host defense by limiting the possibility of the infectious agents to replicate. In the case of SLE, there is a loss of control of these processes with an inadequate clearance of cellular and nuclear debris, which can act as DAMPs, perpetuating the activation of the innate immune system with continuous positive feedback. In addition to apoptosis, other forms of dysfunctional cell death are implicated in SLE pathogenesis, such as pyroptosis mediated by inflammasome activation after recognition by sensor proteins (including NOD-like receptor (NLR)), of pathogen associated molecular patterns (PAMPs) and DAMPs. Once activated, caspase-1 promotes cell death through DNA cleavage, nuclear condensation, plasma membrane pore formation and lysosome exocytosis allowing release into the extracellular space of lysosomal enzymes, partially processed pathogens and autoantigens that being processed by the antigen presenting cells (APCs), stimulate the autoimmune response (Bergsbaken et al., 2011; Magna and Pisetsky, 2015). Netosis, another type of cell death firstly associated with neutrophils, causes the extrusion of nuclear DNA, histones and granular entrapped antimicrobial proteins, called neutrophil extracellular traps (NETs). In SLE, impaired clearance of NETs (like inadequate clearance of apoptotic bodies) is responsible for accumulation of several autoantigens, including self-dsDNA and activation of inflammasome (Villanueva et al., 2011). NETs can also bind another family of pattern recognition receptors (PRRs), the Toll like receptors (TLRs), increasing pro-IL-1β/pro-IL-18 levels and NLRP3 expression (Villanueva et al., 2011). TLR-9 by binding NETs, induces production of type I IFN essential for differentiation of monocytes to DCs that stimulate autoreactive B and T lymphocytes (Lande et al., 2011) and enhance IL-6 and TNF-α release (Torigoe et al., 2018).

SLE can affect any organ system leading to a broad spectrum of clinical manifestation. Serositis is one of lupus related manifestation typically characterized by an increase in acute phase reactants which is a not common occurrence in SLE, unless the concomitant presence of an infectious event. Serositis characterizes other “inflammasome driven” pathologies such as Familial Mediterranean Fever (FMF) and responds promptly to colchicine, a therapy proposed also for the treatment of lupus related serositis.

Colchicine affects several different P2X7R dependent activities through β-tubulin, its primary molecular target, it prevents microtubule polymerization and at high concentrations causes microtubule depolymerization. Colchicine diminishes ATP-induced cationic dye uptake in mouse macrophages, witnessing the requirement of microtubules for P2X7R-dependent pore formation. Moreover, the colchicine interaction with microtubule rearrangement, necessary for IL-1β release, inhibits the ATP-induced P2X7R-dependent release of this cytokine in mouse macrophages (Marques-da-Silva et al., 2011).

The aim of the present study was to explore more deeply the role of the innate immune system in SLE evaluating the P2X7R and NLRP3 expression and activity in SLE patients. Since, serositis is a clinical manifestation associated with inflammatory behavior and one of the most characteristic manifestations of inflammasome-mediated diseases as FMF, the secondary aim was to analyze P2X7R in lupus-related serositis.

Finally, to more extensively investigate the activity of P2X7R, we tested both IL-1β and IL-6, as representative of two different pathways by which this purinergic receptor can mediate inflammatory responses.

## Materials and Methods

### Patients and Study Design

Patients with SLE satisfying the 1997 revised American College of Rheumatology criteria (Hochberg, 1997), were recruited consecutively from the Rheumatology Unit, S. Anna Hospital, University of Ferrara. Clinical (past or ongoing manifestation), serological, demographic characteristics and concomitant therapy were retrospectively recorded. We did a secondary analysis in lupus related serositis. Additional analysis was carried out for the other main clinical manifestations (renal, articular, neurological, and cutaneous) described in the supplementary material. Disease activity and cumulative damage were assessed by SLE disease activity index-2000 (SLEDAI-2K) (Gladman et al., 2002) and the Systemic Lupus International Collaborating Clinics (SLICC) (Gladman et al., 2000), respectively, and recorded in clinical records and dedicated database.

Sero-immunologic tests included: (1) anti-nuclear antibodies (ANA) assessed by indirect immunofluorescence on Hep2 cells (positivity was defined at a titer ≥ 1:160); (2) C3 and C4 dosage measured by nephelometry and hypo-complementaemia was defined by local lab reference values (e.g., C3 < 0.8 and C4 < 0.11 g/l detected in at least two separate occasions); (3) anti-DNA antibodies detected by immunofluorescence on *Crithidia luciliae* (positivity was certified if checked in at least two separate measurements, with a cut-off titer of 1:40); (4) anti-SSA, anti-SSB, anti-Sm, and anti-RNP antibodies detected by immunoblot technique (Line blot, Euroimmun); (5) Lupus anticoagulant (LA) measured accordingly with the recommendation of the Scientific and Standardization Committee of the International Society of Thrombosis and Hemostasis, and anti-CL and anti-beta2GPI measured by enzyme linked immunosorbent assay (ELISA) (Harris et al., 1987). If the result was confirmed in two separate measurements performed 12 weeks apart, positivity for anti-PL and LA was assigned (Horbach et al., 1996). Erythrocyte sedimentation rate (ESR) and C reactive protein (CRP) were measured according to procedures of local laboratory.

Healthy subjects (HC; *n* = 20), volunteers from Ferrara Blood Bank, were considered as control group.

We also analyzed an additional small control-group composed of five patients with an history of serositis affected by diseases other than SLE and excluding FMF (one rheumatoid arthritis and serositis ongoing at the time of sample collection, one systemic sclerosis, one dermatomyositis, one inflammatory bowel disease, and one Sjogren’s syndrome).

All control subjects and patients underwent venous blood collection in EDTA tubes after giving written informed consent. The study was approved by the local ethics committee and conducted in accordance with the amended Helsinki Declaration.

### Separation of Plasma and Peripheral Blood Mononuclear Cells (PBMCs)

Plasma was obtained by centrifugation of blood samples at 1,000 ×*g* for 15 min at 4°C, divided into aliquots and frozen at -80°C until use. The remaining cellular component was used to separate peripheral blood mononuclear cells (PBMCs) by stratification on a Ficoll (Health Care, Uppsala, Sweden) gradient as previously described (Falzoni et al., 1995). PBMCs were divided into three aliquots of 2 × 10^6^ cells each, and used for: (1) setting up short-term cell cultures, (2) measurement of calcium fluxes in fluorimetry, and (3) RNA extraction.

### Preparation and Stimulation of PBMCs Short-Term Cultures

Peripheral blood mononuclear cells were suspended at the concentration of 5×10^5^ cells/ml of 10% FBS supplemented RPMI (Gibco, Thermo Scientific, Waltham, MA, United States) without antibiotics and placed into 12-well plates for cell culture, using four wells for each patient. After an overnight incubation at 37°, 5% CO_2_, non-adherent cells were removed and the adherent mononuclear cells (90% macrophages, as verified detecting shape modification at optical microscopy) were incubated in 10% FBS-supplemented RPMI under the following conditions: (1) no additions (control) for 5 h; (2) 1 μg/ml lipopolysaccharide (LPS) (Sigma-Aldrich, St. Louis, MO, United States) for 4 h; (3) 1 μg/ml LPS for 4 h, followed by 300 μM BzATP (Sigma-Aldrich) for 1 h; (4) none for 4 h, followed by 300 μM BzATP for 1 h. At the end, the supernatants were collected, centrifuged and frozen at -80°C until use.

### IL-1β and IL-6 ELISA

IL-1β and IL-6 concentrations in both plasma and PBMCs supernatants were measured by ELISA using the human IL-1β/IL-1F2 and the human IL-6 Quantikine ELISA kits (R&D System, Bio-Techne, Minneapolis, MN, United States), respectively, following manufacturer’s instructions. Standard and samples were tested in duplicate. Optical density was detected using a Thermo Scientific Multiskan FC plate reader. Cytokines concentrations were expressed as pg/ml.

### Measurement of Intracellular Ca^2+^ Concentrations [Ca^2+^]_i_

Variations of intracellular Ca^2+^ concentrations [Ca^2+^]_i_ were measured using the Fura-2/AM (Sigma-Aldrich) fluorescent probe. For this 2×10^6^ PBMCs were loaded with 2 μM Fura-2/AM for 20 min at 37°C. After washes, PBMCs were employed for the assay at 37°C in a Cary Eclipse Fluorescence Spectrophotometer (Agilent Technologies, Cernuso SN, Milan, Italy) using 500 μM BzATP as stimulus. In some experiments, PBMCs were pre-incubated for 1 h at 37°C in 5% CO_2_ with 20–200 μM chloroquine (Sigma-Aldrich), washed and tested for variation of [Ca^2+^]_I_ as above.

### RNA Extraction

Peripheral blood mononuclear cells were suspended in Trizol (Thermo Fisher Scientific, Waltham, MA, United States) and RNA extracted using the PureLink RNA Mini Kit (Thermo Fisher Scientific) following manufacturer’s instructions. RNA was suspended in RNAse free water and its concentration was determined using a NanoDrop 2000 spectrophotometer (Thermo Fisher Scientific).

#### RT-PCR

RNA retro-transcription was performed using the High Capacity cDNA reverse transcription kit (Thermo Fisher Scientific) following manufacturer’s instructions. RT-PCR for P2X7R, NLRP3, and GAPDH as endogenous control was performed using an RT-PCR kit (Thermo Fisher Scientific) in a PCR Biometra UNO- Thermoblock (DASIT, Cornaredo, Milan, Italy) using the following primers: Human p2x7 assay ID: Hs00175721_m1, Human NLRP3 assay ID: Hs00366465_m1. Pre-developed taqman endogenous control human GAPDH code 4326317E was used as housekeeping gene.

### Statistical Analysis

For descriptive analysis, discrete variables were expressed as absolute and relative frequencies while continuous variables as mean ± standard error (SE). The comparison between the groups was performed with the chi-squared test, *t*-test or Mann–Whitney and Wilcoxon test for non-parametric variables. The correlation index was used to evaluate the relationship between continuous variables, the correlation Spearman’s ranks index was used, where indicated. Analyses were performed using Graphpad version 6.

## Results

### Clinical Characteristics

A total of 48 SLE patients (45 women and 3 men) were enrolled, 16 (33.3%) of which had a history of serositis (previous or ongoing at the time of blood collection). The mean age (mean ± SD) of SLE population was of 41.9 ± 10.2 years, disease duration of 130.6 ± 96.7 months, the mean SLEDAI-2K 4.2 ± 4.4 and the mean SLICC equal to 0.6 ± 0.8. Demographic, clinical and pharmacologic treatments of all patients were collected (Table 1). No significant difference in clinical characteristics between patients with (SLE-S) or without (SLE-NS) a history of serositis was found, except for CRP, which was significantly higher in SLE-S (Table 2).

**Table 1:** Demographic, clinical features and pharmacologic treatments of the SLE patients.

	SLE patients (*n* = 48)
**Demographic parameters**	
No. female/male	45 (93.7%)/3 (6.3%)
Age (mean ± SD) (years)	41.9 ± 10.2
Disease duration (months)	130.6 ± 96.7
**Past clinical pattern, n° (%)**	
Cutaneous (acute/subacute/chronic)	37 (77.1%)
Articular	31 (64.6%)
Serositis	16 (33.3%; three ongoing) type of serositis: • 4 pericardial • 7 pleural • 5 both pleural + pericardial
Renal	12 (25%) → glomerulonephritis histological class: • 1 both II and III class • 2 III class • 1 both III and V class • 5 IV class • 1 both IV and V class • 2 V class
Neurological	9 (18.8%) → type of neurological involvement: • 6 ischemic lesions • 1 headache • 1 depression • 1 myasthenia
Anemia	6 (12.5%)
Leukopenia	17 (35.4%)
Thrombocytopenia	3 (6.25%)
**Serological parameters n°(%)**	
ANA positivity	48 (100%)
ENA positivity	31 (64.6%)
Anti-DNA positivity	40 (83.3%)
Hypocomplementemia	40 (83.3%)
aPL (aCL, β2GPI, and/or LA) positivity	15 (31.2%)
**Ongoing treatment**	
Hydroxychloroquine (200 mg/day)	38 (79.2%)
Corticosteroids (2.5 up to 12.5 mg/day)	40 (83.4%)
Mycophenolate mofetil	8 (16.7%)
Cyclosporine A	2 (4.2%)
Azathioprine	4 (8.3%)
Methotrexate (10–15 mg/week)	3 (6.3%)
PEX	1 (2.1%)
Rituximab	1 (2.1%)
Belimumab	1 (2.1%)
**Cumulative dosage of steroids gr (mean ± SD)**	19.8 ± 18.0
**Ongoing clinical manifestation n° (%)**	
Serositis	3 (6.25%)
Arthralgia	6 (12.5%)
Cutaneous manifestation	6 (12.5%)
Renal (proteinuria > 500 mg/24 h)	5 (10.4%)
Neurological	0
Leukopenia	4 (8.34%)
**Current serological parameters**	
Anti-DNA	40 (83.3%)
Hypocomplementemia	40 (83.3%)
ESR (mm/h) (mean ± SD) (years)	16.4 ± 9.3
CRP (mean ± SD) (years)	0.7 ± 1.6
**Current clinimetric parameters**	
SLEDAI-2K (mean ± SD)	4.2 ± 4.4
SLICC (mean ± SD)	0.6 ± 0.8


*SLEDAI-2K, SLE disease activity index-2000; SLICC, SLE International Collaborating Clinics Damage Index; aPL, antiphospholipid antibodies including anti-cardiolipin antibodies (aCL) and lupus anticoagulant (LA); β2GPI, beta-2 glycoprotein 1 antibodies; ENA, extractable nuclear antigens antibodies; PEX, plasma exchange; ESR, erythrocyte sedimentation rate; CRP, C reactive protein.*

**Table 2:** Comparison between patients with a positive history of serositis (SLE-S) vs. patients without history of serositis (SLE-NS).

	SLE-NS n° (%)	SLE-S (n°; %)	p (SLE-NS vs. SLE-S)
**No. of patients**	**32 (66.7%)**	**16 (33.3%)**	
**Demographic parameters**			
No. female/male	31/1	14/2	0.25
Age (mean ± SD) (years)	40.8 ± 8.4	44.2 ± 13.1	0.28
Disease duration (months)	134.7 ± 98.3	122.5 ± 96.2	0.68
**Ongoing treatment**			
Hydroxychloroquine (200 mg/day)	25 (78.1%)	13(81.2%)	1
Corticosteroids (2.5 up to 12.5 mg/day)	26 (81.3%)	6 (18.8%)	0.70
Mycophenolate mofetil	7 (21.9%)	1 (6.25%)	0.24
Cyclosporine A	2 (6.3%)	0	0.55
Azathioprine	2 (6.25%)	2(12.5%)	0.59
Methotrexate (10–15 mg/week)	1 (3.3%)	2 (15.38%)	0.21
PEX	0	1 (6.25%)	0.33
Rituximab	1 (3.1%)	0	1
Belimumab	0	1 (6.25%)	0.33
**Ongoing dosage of steroids mg (mean ± SD)**	4.3 ± 3.6	5.3 ± 4.5	0.42
**Cumulative dosage of steroids gr (mean ± SD)**	21.6 ± 20.6	18.9 ± 16.8	0.64
**Current serological parameters**			
Anti-DNA	20 (62.5%)	11 (68.8%)	1.00
Hypocomplementemia	13 (40.63%)	11 (68.8%)	0.125
ESR (mm/h) mean ± SD years	15.4 ± 1.5	18 ± 2.7	0.29
CRP mean ± SD years	0.4 ± 0.02	1.4 ± 0.67	0.04
**Current clinimetric parameters**			
SLEDAI-2K (mean ± SD)	3.8 ± 4.2	4.9 ± 4.6	0.42
SLICC (mean ± SD)	0.45 ± 0.8	0.8 ± 0.9	0.17


*SLEDAI-2K, LE disease activity index-2000; SLICC, SLE International Collaborating Clinics Damage Index; aPL, antiphospholipid antibodies including anti-cardiolipin antibodies (aCL) and lupus anticoagulant (LA); β2GPI, beta-2 glycoprotein 1 antibodies; ENA, extractable nuclear antigens antibodies; PEX, plasma exchange; ESR, erythrocyte sedimentation rate; CRP, C reactive protein. Chi-square, *t*-test or Mann–Whitney for non-parametric data.*

### Evaluation of Plasma IL-1β and IL-6

**FIGURE 1:**
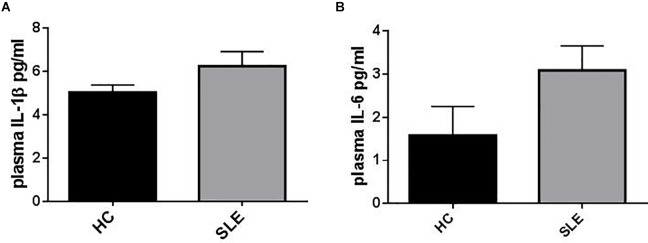
Representation of IL-1β and IL-6 plasma levels in patients (SLE) vs. healthy control (HC). **(A)** Plasma IL-1β levels were not statistically different between HC and SLE (SLE vs. HC = 6.24 ± 0.61 vs. 5.42 ± 0.384, *p* = 0.26). Unpaired *t*-test. **(B)** Plasma IL-6 levels resulted higher in SLE vs. HC (SLE vs. HC = 3.09 ± 0.57 vs. 1.58 ± 0.67, *p* = 0.191). Unpaired *t*-test. Data are means ± SE.

**FIGURE 2:**
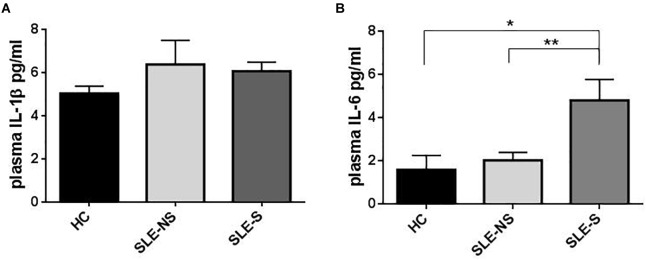
Representation of IL-1β and IL-6 plasma levels in patients with serositis (SLE-S), without serositis (SLE-NS) compared to healthy control (HC). **(A)** Plasma IL-1β levels were not statistically significant between HC, SLE-S, and SLE-NS (SLE-NS vs. HC = 6.39 ± 1.13 vs. 5.42 ± 0.384, *p* = 0.45), (SLE-S vs. HC = 6.07 ± 0.38 vs. 5.42 ± 0.38, *p* = 0.246), (SLE-S vs. SLE-NS = 6.07 ± 0.38 vs. 6.39 ± 1.13, *p* = 0.80). Unpaired *t*-test. **(B)** Plasma IL-6 levels resulted significantly higher in SLE-S compared to both HC and SLE-NS: (SLE-NS vs. HC = 2.02 ± 0.37 vs. 1.58 ± 0.67, *p* = 0.54), (SLE-S vs. HC = 4.8 ± 0.97 vs. 1.58 ± 0.67, *p* = 0.037), (SLE-S vs. SLE-NS 4.8 ± 0.97 vs. 2.02 ± 0.37, *p* = 0.009). Unpaired *t*-test. Data are means ± SE. Only significant differences are shown. ^∗^*p* < 0.05, ^∗∗^*p* < 0.01.

Plasma IL-1β levels did not differ significantly between patients (SLE) and HC (Figure 1A) and a sub-analysis performed in SLE-S and SLE-NS gave similar results (Figure 2A). In a small population (*n* = 5) of patients with serositis without SLE, plasma IL-1β levels were significantly higher than in HC subjects (Supplementary Figure 1A), and even higher than SLE-S (Supplementary Figure 1B). Plasma IL-6 levels resulted higher, although not significantly, in SLE patients (3.09 ± 0.57 pg/ml) compared with (1.58 ± 0.67 pg/ml) (*p* = 0.191) (Figure 1B). Subanalysis of SLE patients group showed that plasma IL-6 was significantly higher in SLE-S (4.8 ± 0.97 pg/ml) vs. both SLE-NS (2.02 ± 0.37 pg/ml, *p* = 0.009) and HC (1.58 ± 0.67 pg/ml, *p* = 0.037) (Figure 2B). Subjects with serositis without SLE, showed plasma IL-6 levels higher than SLE subjects (Supplementary Figures 1C,D).

### Evaluation of IL-1β and IL-6 in Cultured Macrophages Supernatants

**FIGURE 3:**
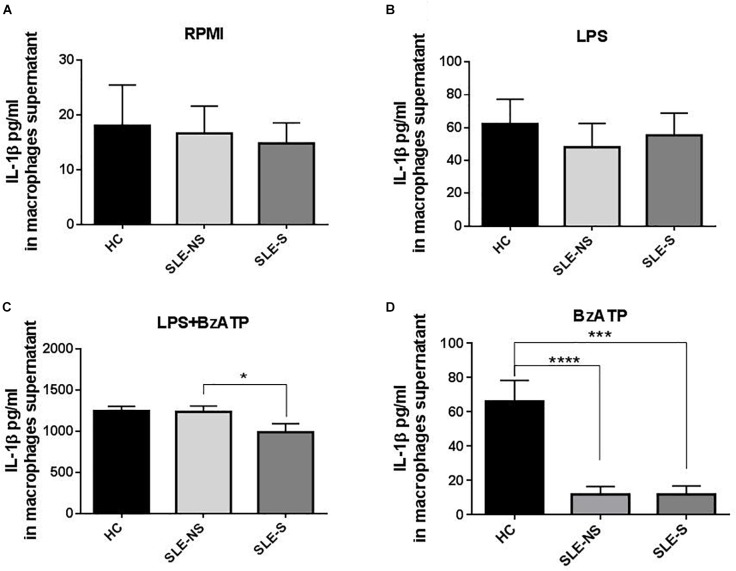
Representation of IL-1β levels in macrophages supernatants from patients with serositis (SLE-S), without serositis (SLE-NS) and healthy controls (HC). **(A)** IL-1β released from unstimulated macrophages maintained for 5 h in 10% FBS supplemented RPMI was not significantly different between HC and SLE patients (SLE-NS vs. HC = 16.63 ± 5 vs. 18.05 ± 7.41, *p* = 0.88; SLE-S vs. HC = 14.82 ± 3.76 vs. 18.05 ± 7.41, *p* = 0.67) and between SLE-S vs. SLE-NS (*p* = 0.80). Unpaired *t*-test. **(B)** IL-1β released from macrophages stimulated for 4 h with 1 μg/ml LPS in 10% FBS-supplemented RPMI was not significantly different between HC and SLE patients (SLE-NS vs. HC = 48.11 ± 14.53 vs. 62.28 ± 15.09, *p* = 0.556; SLE-S vs. HC = 55.38 ± 13.47 vs. 62.28 ± 15.09, *p* = 0.736), and between SLE-S vs. SLE-NS (*p* = 0.745). Unpaired *t*-test. **(C)** IL-1β released from macrophages stimulated for 4 h with 1 μg/ml LPS and for 1 h with 500 μM BzATP in 10% FBS-supplemented RPMI was not different between HC and SLE patients (SLE-NS vs. HC = 1237 ± 70.39 vs. 1246 ± 58.61, *p* = 0.936; SLE-S vs. HC = 988.7 ± 103.6 vs. 1246 ± 58.61, *p* = 0.072). IL-1β was significantly lower in SLE-S compared to SLE-NS (*p* = 0.0476). Unpaired *t*-test. **(D)** IL-1β released from macrophages stimulated for 1 h with 300 μM BzATP in 10% FBS-supplemented RPMI was significantly lower in SLE patients respect to HC (SLE-NS vs. HC = 63.91 ± 18.94 vs. 208.4 ± 34.87, *p* < 0.0006; SLE-S vs. HC = 59.29 ± 24.02 vs. 208.4 ± 34.87, *p* = 0.0015). IL-1β was not different between SLE-S vs. SLE-NS (*p* = 0.881). Unpaired *t*-test. Data are means ± SE. Only significant differences are shown. ^∗^*p* < 0.05, ^∗∗∗^*p* < 0.005, ^∗∗∗∗^*p* < 0.001.

**FIGURE 4:**
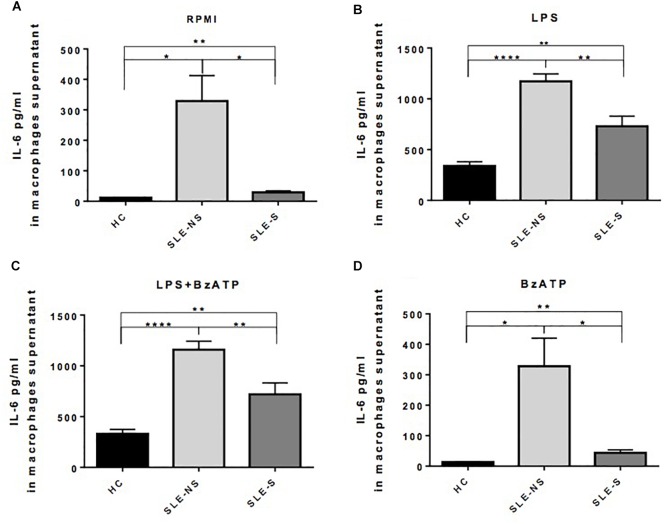
Representation of IL-6 levels in macrophages supernatants from patients with serositis (SLE-S), without serositis (SLE-NS) and healthy controls (HC). **(A)** IL-6 released from unstimulated macrophages maintained for 5 h in 10% FBS supplemented RPMI was significantly higher in SLE patients vs. HC (SLE-NS vs. HC: 329.0 ± 83.74 vs. 11.83 ± 0.51, *p* = 0.0102; SLE-S vs. HC = 29.18 ± 4.85 vs. 11.83 ± 0.51, *p* = 0.0021) and in patients without serositis vs. patients with serositis (SLE-S vs. SLE-NS, *p* = 0.014). **(B)** IL-6 released from macrophages stimulated for 4 h with 1 μg/ml LPS in 10% FBS-supplemented RPMI was significantly higher in SLE patients vs. HC (SLE-NS vs. HC: 1172 ± 74.25 vs. 339.9 ± 41.37, *p* < 0.0001; SLE-S vs. HC = 728.8 ± 101.2 vs. 339.9 ± 41.37, *p* = 0.002) and in patients without serositis vs. patients with serositis (SLE-S vs. SLE-NS, *p* = 0.001). **(C)** IL-6 levels from macrophages stimulated for 4 h with 1 μg/ml LPS, and for 1 h with 300 μM BzATP in 10% FBS-supplemented RPMI were significantly higher in SLE patients vs. HC (SLE-NS vs. HC: 1159 ± 83.87 vs. 330.7 ± 43.46, *p* < 0.0001; SLE-S vs. HC = 719.7 ± 112.6 vs. 330.7 ± 43.46, *p* = 0.004) and in patients without serositis vs. patients with serositis (SLE-S vs. SLE-NS, *p* = 0.003). **(D)** IL-6 released from macrophages stimulated for 1 h with 300 μM BzATP in 10% FBS-supplemented RPMI was significantly higher in SLE patients vs. HC (SLE-NS vs. HC: 328 ± 92.1 vs. 13.38 ± 0.76, *p* = 0.019; SLE-S vs. HC = 43.95 ± 9.49 vs. 13.38 ± 0.76, *p* = 0.005) and in patients without serositis vs. patients with serositis (SLE-S vs. SLE-NS, *p* = 0.028). Data are means ± SE. Only significant differences are shown. ^∗^*p* < 0.05, ^∗∗^*p* < 0.01, ^∗∗∗∗^*p* < 0.001.

Release of IL-1β from macrophages maintained for 5 h in RPMI or stimulated for 4 h with 1 μg/ml LPS was not significantly different between SLE and HC (pg/ml; mean ± SE) (Figure 3A,B). Stimulation of macrophages with 300 μM BzATP for 1 h following LPS treatment provoked IL-1β release significantly lower in SLE-S compared to SLE-NS (988.7 ± 103.6 vs. 1237 ± 70.39; *p* = 0.048) whereas no significant difference was found with HC (Figure 3C). The most relevant difference between SLE and HC was visible when macrophages were stimulated with 300 μM BzATP alone (Figure 3D). In this case IL-1β release was significantly lower in SLE patients respect to HC (SLE-NS vs. HC = 11.84 ± 4.68 vs. 66.19 ± 12.31, *p* < 0.0001; SLE-S vs. HC = 11.95 ± 4.99 vs. 66.19 ± 12.31, *p* = 0.0002) without difference between patients with serositis vs. patients without serositis (SLE-S vs. SLE-NS, *p* = 0.987). Patients with serositis without SLE, showed IL-1β release after stimulation with BzATP significantly higher respect to both SLE-S and SLE-NS (Supplementary Figure 2D). IL-1β released from unstimulated macrophages and after stimulation with LPS alone was not significantly different between HC, SLE patients and serositis patients (Supplementary Figures 2A,B). IL-6 was found significantly higher in supernatants of macrophages from SLE compared to HC in all different experimental conditions (pg/ml; mean ± SE) (Figure 4). IL-6 released from unstimulated macrophages maintained for 5 h in RPMI was significantly higher in SLE patients vs. HC (SLE-NS vs. HC: 329.0 ± 83.74 vs. 11.83 ± 0.51, *p* = 0.0102; SLE-S vs. HC = 29.18 ± 4.85 vs. 11.83 ± 0.51, *p* = 0.0021) and in patients without serositis vs. patients with serositis (SLE-NS vs. SLE-S, *p* = 0.014) (Figure 4A). The release of IL-6 from macrophages stimulated for 4 h with 1 μg/ml LPS was significantly higher in SLE patients vs. HC (SLE-NS vs. HC: 1172 ± 74.25 vs. 339.9 ± 41.37, *p* < 0.0001; SLE-S vs. HC = 728.8 ± 101.2 vs. 339.9 ± 41.37, *p* = 0.002) and in patients without serositis vs. patients with serositis (SLE-NS vs. SLE-S, *p* = 0.001) (Figure 4B). Stimulation for 4 h with 1 μg/ml LPS, and for 1 h with 300 μM BzATP produced a significantly higher IL-6 release in SLE patients vs. HC (SLE-NS vs. HC: 1159 ± 83.87 vs. 330.7 ± 43.46, *p* < 0.0001; SLE-S vs. HC = 719.7 ± 112.6 vs. 330.7 ± 43.46, *p* = 0.004) and in patients without serositis vs. patients with serositis (SLE-NS vs. SLE-S, *p* = 0.003) (Figure 4C). Similarly, IL-6 released following macrophage stimulation for 1 h with BzATP was significantly higher in SLE patients vs. HC (SLE-NS vs. HC: 328 ± 92.1 vs. 13.38 ± 0.76, *p* = 0.019; SLE-S vs. HC = 43.95 ± 9.49 vs. 13.38 ± 0.76, *p* = 0.005) and in patients without serositis vs. patients with serositis (SLE-NS vs. SLE-SS, *p* = 0.028) (Figure 4D).

IL-6 released from macrophages of patients with serositis without SLE was significantly lower than that secreted by macrophages of SLE-NS when stimulated by LPS (Supplementary Figure 3B) while was significantly higher than that secreted by macrophages of HC following LPS+BzATP stimulation (Supplementary Figure 3C). No significant difference of IL-6 levels were found in patients with serositis after stimulation by RPMI and BzATP alone (Supplementary Figures 3A–D).

### Evaluation of Variations of Intracellular Ca^2+^ Concentration (Δ[Ca^2+^]_i_) With Fura-2/AM in PBMCs After BzATP Stimulation

**FIGURE 5:**
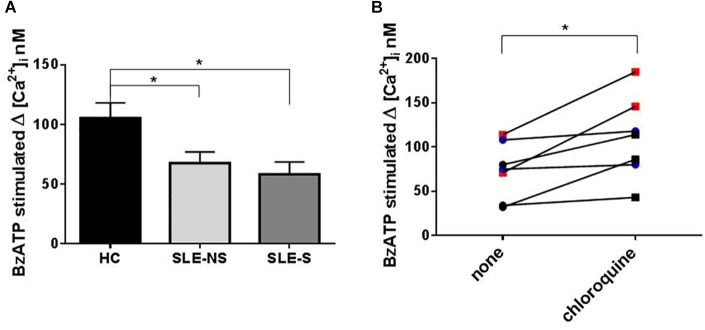
Evaluation of Calcium influx with Fura-2 in PBMCs after BzATP stimulation and chloroquine pre-treatment. **(A)** The increase of intracellular Ca^2+^ concentration (Δ[Ca^2+^]_i_) following stimulation with 500 μM BzATP was significantly lower in PBMCs from patients (SLE) vs. controls (HC) (SLE-NS vs. HC = 67.69 ± 9.23 vs. 105.5 ± 12.75, *p* = 0.0228; SLE-S vs. HC = 58.25 ± 10.28 vs. 105.5 ± 12.75, *p* = 0.0147). Unpaired *t*-test. Only significant differences are shown. **(B)** The effect of 200 mM chloroquine on Δ[Ca^2+^]_i_ was evaluated on PBMCs from four controls and three patients. Each line represents a patients or a control subject. The Δ[Ca^2+^]_i_ was higher after chloroquine treatment respect to basal conditions (Wilcoxon matched-pairs signed rank test, *p* = 0.016) both in controls and patients (pre vs. post treatment: 92.0 ± 11.07 vs. 132.3 ± 22.18, *p* = 0.13) and patients (48.67 ± 15.68 vs. 81 ± 20.65, *p* = 0.25). Data are means ± SE. ^∗^*p* < 0.05.

Variation of intracellular Ca^2+^ concentration (Δ[Ca^2+^]_i_ (Fura-2/AM) nM ± SE) after stimulation of PBMCs with 500 μM BzATP, a direct measurement of P2X7R activity, was significantly lower in SLE than in HC (SLE vs. HC = 64.1 ± 6.84 vs. 105.5 ± 12.75, *p* = 0.008; SLE-NS vs. HC = 67.69 ± 9.23 vs. 105.5 ± 12.75; *p* = 0.0267; SLE-S vs. HC = 58.25 ± 10.28 vs. 105.5 ± 12.75, *p* = 0.0215), with no significant difference between patients without serositis vs. patients with serositis (SLE-NS vs. SLE-S, *p* = 0.674) (Figure 5A). PBMCs from five patients with serositis without any evidence of SLE, showed a BzATP-stimulated increase of [Ca^2+^]_i_ corresponding to 191.8 ± 50.92, significantly higher than that measured in all the other groups of subjects (serositis vs. HC *p* = 0.008; serositis vs. SLE-NS *p* = 0.0005; serositis vs. SLE-S *p* = 0.0016) (Supplementary Figure 4).

To evaluate the effect of chloroquine on P2X7R activity, PBMCs from four controls and three patients underwent an evaluation of Ca^2+^ influx in normal conditions and after treatment for 1 h with 200 μM chloroquine. The Δ[Ca^2+^]_i_ was higher after chloroquine treatment respect to basal conditions (Wilcoxon matched-pairs signed rank test *p* = 0.016) both in controls and patients (pre vs. post treatment: 92.0 ± 11.07 vs. 132.3 ± 22.18; *p* = 0.13) and patients (48.67 ± 15.68 vs. 81 ± 20.65; *p* = 0.25) (Figure 5B).

### Evaluation of P2X7R and NLRP3 Expression With RT-PCR

**FIGURE 6:**
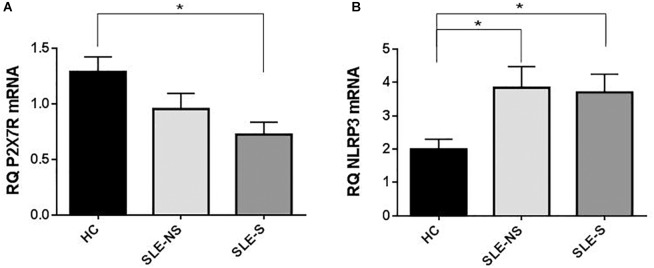
Expression of P2X7R and NLRP3 mRNA in PMBCs from patients (SLE) vs. healthy control (HC). **(A)** P2X7R was significantly less expressed in SLE vs. HC (SLE vs. HC = 0.87 ± 0.10 vs. 1.29 ± 0.13, *p* = 0.02), particularly in patients with serositis (SLE-S vs. HC = 0.724 ± 0.11 vs. 1.29 ± 0.13, *p* = 0.019). **(B)** NLRP3 expression resulted significantly higher in SLE vs. HC (SLE vs. HC = 3.80 ± 0.46 vs. 1.99 ± 0.30, *p* = 0.018). Data are means ± SE. Only significant differences are shown. ^∗^*p* < 0.05.

At RT-PCR, P2X7R resulted significantly less expressed in PBMCs of patients compared to controls (mean ± SE) (SLE vs. HC = 0.87 ± 0.1 vs. 1.29 ± 0.13; *p* = 0.02), particularly in patients with serositis (SLE-S vs. HC = 0.724 ± 0.11 vs. 1.29 ± 0.13; *p* = 0.019) (Figure 6A). On the contrary, NLRP3 expression resulted significantly higher in PBMCs of patients than in controls (SLE vs. HC = 3.80 ± 0.46 vs. 1.99 ± 0.30; *p* = 0.018) also considering the two sub-categories separately (SLE-NS vs. HC = 3.846 ± 0.633 vs. 1.995 ± 0.302, *p* = 0.013; SLE-S vs. HC = 3.702 ± 0.552 vs. 1.995 ± 0.302, *p* = 0.036) (Figure 6B).

### Evaluation of the Role of Clinical Factors on P2X7R Activity Evaluated as Δ[Ca^2+^]_i_ (Fura-2)

No significant correlation between disease activity/disease duration and P2X7R activity assessed by Ca^2+^ influx measurement was detected (Table 3). For the evaluation of the effect of therapy, we have collected all patients taking immunosuppressive therapy in a single group called “ongoing major immunosuppressive therapy.” We also assessed separately the influence of ongoing hydroxychloroquine and of ongoing steroids therapy (Table 3). Finally, the effect of steroid dosage (current or cumulative) was assessed (Table 3). No significant influence of therapy on Δ[Ca^2+^]_i_ was detected.

**Table 3:** Correlation between disease activity, disease duration, and corticosteroids dosage with P2X7R activity.

	SLEDAI	Disease duration	Cumulative dosage of steroids	Current dosage of steroids	Ongoing hydroxychloroquine (38 patients)	Ongoing steroids (40 patients)	Ongoing major immunosuppressive therapy (18 patients)
	Spearman rho; *p*	rho; *p*	Spearman rho; *p*	Spearman rho; *p*	Yes vs. No; *p*	Yes vs. No; *p*	Yes vs. No; *p*
ΔCa^2+^ (Fura-2) nM (±SD)	–0.4; *p* = 0.1	0.08; *p* = 0.73	0.18; *p* = 0.47	–0.13; *p* = 0.61	70.8 ± 30.8 vs. 53.6 ± 34.2; *p* = 0.32	66.3 ± 33.9 vs. 61.5 ± 4.9; *p* = 0.84	53.8 ± 37.7 vs. 76.2 ± 22.6; *p* = 0.15


*Spearman correlation.*

## Discussion

The aim of our study was to evaluate the expression and the activity of P2X7R and NLRP3 inflammasome in a cohort of patients with SLE, analyzing more deeply the cases that presented a history of serositis, a clinical manifestation that more than others recalls auto-inflammatory diseases such as FMF. A sub-analysis performed on SLE patients showed no differences between SLE-NS and SLE-S as regard clinical parameters, disease activity, demographic characteristics, and treatment, except the CRP levels (Table 2).

Evidence in both mouse models and humans had revealed a possible role for P2X7R and NLRP3 inflammasome in SLE pathogenesis. MRL/lpr mice showed increased renal expression of P2X7R, NLRP3, ASC and caspase 1, resulting in increased production of IL-1β and IL-18 and treatment with the P2X7R antagonist Brilliant Blue G reduced proteinuria, serum anti-DNA antibodies and, at renal histology, glomerular cellularity, signs of vasculitis and IgG and C3 deposition (Zhao et al., 2013). Also in lupus-like nephritis induced by intraperitoneal injection of pristane, caspase-1-/- mice presented reduction of autoantibodies (anti-DNA and anti-RNP) and hypergammaglobulinemia compared to pristane-treated wild type mice (Peairs et al., 2009). In humans, a study on renal biopsies showed increased P2X7R expression in lupus patients compared to controls (Turner et al., 2006) and increased IL-1β serum levels in SLE patients has been demonstrated (Cigni et al., 2015).

Contrary to these previous observations, our study does not reveal a direct role of P2X7R as an inducer of the inflammatory response in patients with SLE. P2X7R expression (as mRNA) (Figure 6) and activity (as BzATP Δ[Ca^2+^]_i_) (Figure 5) were indeed reduced in PBMCs from SLE patients respect to those from HC. In addition, BzATP-stimulated IL-1β release, from macrophages of SLE patients was reduced respect to controls (Figure 3). In our patients, plasma IL-1β levels were only slightly, not significantly increased compared to healthy subjects and cultured macrophages from these patients released substantially similar levels of IL-1β after stimulation with LPS alone or followed by BzATP. These results suggest that P2X7R reduced expression and activity in SLE patients might be partially compensated by increased expression of NLRP3, as revealed by RT-PCR (Figure 6). The P2X7R defect in SLE patients was underlined by the highest difference between controls and patients in IL-1β release when macrophages were stimulated by BzATP alone which can be considered a powerful P2X7R agonist (Figure 3D).

Since P2X7R activation is also implicated in the production of other inflammatory cytokines such as IL-1α, TNF-α, and IL-6, independently of the NLRP3 inflammasome (Gicquel et al., 2015), to more extensively investigate the P2X7R activity we also tested IL-6 levels as representative of a different pathway by which P2X7R can support inflammatory responses. Plasma IL-6 levels were higher in SLE subjects, especially in SLE-S subgroup (Figure 1B, 2B). Macrophages from SLE patients released significantly higher levels of IL-6 *in vitro*, even in basal conditions and after stimulation with LPS and/or BzATP (Figure 4). IL-6 release was particularly increased in the SLE-NS sub-group suggesting that macrophages from SLE-S patients might be de-sensitized to IL-6 production *in vitro*. In SLE, IL-6 levels seem not to be correlated to an inflammatory state as suggested by the lack of correlation between CRP and IL-6 levels (both in plasma and in supernatants) (Supplementary Table 6). These results lead us to hypothesize that in our SLE patients a pathogenetic pathway resulting in increased production of IL-6, would prevail on the IL-1β pathway mediated by P2X7R that indeed appears downregulated. IL-6 release could be independent of the P2X7R-NLRP3 axis. The inflammasome may indeed be activated by different pathways stimulating other PRRs, such as TLRs able not only to promote IL-1β and IL-18 gene transcription, but also to increase NLRP3 activity and IL-6 production (Villanueva et al., 2011; Klonowska-Szymczyk et al., 2017).

To ascertain that the observed data were associated not only to serositis but more generally to SLE, a sub-analysis performed in other major lupus-related clinical manifestations (nephritis, arthritis, skin, and neuropsychiatric involvement) excluded the association with the activity and expression of P2X7R, NLRP3 and production of IL-1 β and IL-6 (see Supplementary Tables 1–4). The analysis carried out on the group of patients with serositis, suffering from pathologies other than SLE and FMF, showed that P2X7R was more active in these subjects respect to SLE patients, as evidenced by increased IL-1β in both plasma and supernatants of macrophages after stimulation with pure agonist BzATP (Supplementary Figures 1A, 2D) and augmented BzATP Δ[Ca^2+^]_I_ increase (Supplementary Figure 4). These results, despite the limitations of the small sample size (five patients with serositis), reinforce our hypothesis that P2X7R is downregulated in patients with SLE, regardless of the type of clinical manifestation.

Cytokine production is not the only activity mediated by P2X7R. In case of pore formation following prolonged stimulation with ATP, P2X7R could mediate a cytotoxic effect promoting cell death. A reduced expression of P2X7R would be implicated in defective apoptosis of different cellular types like T follicular helper (Tfh) cells in germinative centers with consequent enhanced activation of B lymphocytes (Perruzza et al., 2017). A study conducted on 42 SLE patients, showed that enhanced expansion of Tfh cells was correlated with diminished cell death mediated by P2X7R (Gualtierotti et al., 2017; Faliti et al., 2019). In accordance with this evidence, reduced expression of P2X7R detected in our study might be implicated in SLE pathogenesis through a different mechanism mediated by its role in cellular growth control. Further studies will be needed to clarify the role of P2X7R in SLE, and especially if the reduced expression of the receptor has a major role in SLE pathogenesis or if this finding could be considered a consequence of the disease itself. In presence of a persistent inflammatory state as in SLE (consistent with elevated IL-6 levels found in the patients of this study), there is an increase in ATP levels in the extracellular microenvironment able to activate P2X7R. An adaptation mechanism, secondary to the chronic inflammatory state, could, therefore, induce a negative feedback effect on P2X7R expression. On the other side, the increase in IL-6 could represent an activated pathway in response to a primary P2X7R downregulation. The role of IL-6 in SLE has been evaluated in the pathogenesis of different clinical manifestations. For example, it has been detected in serum and urine of SLE patients with lupus nephritis (Peterson et al., 1996) and in cerebrospinal fluid in patients with neuropsychiatric manifestations (Yoshio et al., 2016), especially in presence of cognitive impairment (Wiseman et al., 2017). These evidences would make reasonable an attempt of targeting IL-6 in SLE. A phase I study evaluated the effect of high-affinity human anti-IL-6 monoclonal antibody sirukumab in SLE patients with LN of class III and IV that have already received induction therapy with mycophenolate mophetil (MMF) or cyclophosphamide and presented significant proteinuria despite maintenance treatment with MMF or azathioprine. After 6 months of treatment (21 patients treated with sirukumab and 4 with placebo) no significant improvement in proteinuria and disease activity was found after adding the anti-IL-6 treatment to immunosuppressive therapy (Rovin et al., 2016). Another phase II study in SLE patients with the humanized monoclonal antibody tocilizumab against the chain of the IL-6 receptor, showed a reduction of auto-antibodies production and plasma-cells and significant clinical improvement especially in case of arthritis and fatigue (Illei et al., 2010). Finally a case report demonstrated the efficacy of tocilizumab in lupus serositis (Kamata and Minota, 2012; Ocampo et al., 2016).

Moreover, also IL-1β and P2X7R-inflammasoma axis, were considered possible therapeutic targets in other reports. Anakinra, an IL-1β receptor antagonist, was for example shown to be effective and safe in a small study conducted on SLE patients with refractory arthritis (Ostendorf et al., 2005). Inflammation parameters (ESR and CRP) are generally altered in the SLE during few conditions like infection or serositis (Ryu et al., 2017). In addition, this manifestation responds promptly to colchicine, a treatment generally used in diseases considered “inflammasome guided” as the FMF (Morel et al., 2015; Padeh and Berkun, 2016). The primary molecular target of colchicine is β-tubulin inducing microtubule de-polymerization. In mouse macrophages the lack of microtubule rearrangement provoked by colchicine, affected several different P2X7R activities such as pore formation and IL-1β release (Marques-da-Silva et al., 2011). These aspects made us to hypothesize that P2X7R-inflammasome axis could be a fundamental actor in the pathogenesis of lupus serositis which is usually successfully treated with colchicine.

Our study has many limitations: first, the patients enrolled had a prolonged disease duration (over 10 years) so they have been subjected to a long period of treatment. The majority of patients did not present active disease expressed in term of SLEDAI-2K (4.2 ± 4.4). Considering the serological domains of SLEDAI-2K, 83.3% of patients had both anti-DNA positivity and hypocomplementemia and consequently, a limited sample size presented an active clinical manifestation at the enrollment. To assess the influence of the presence of an active manifestation on P2X7R activity and expression, we compared patients in clinical remission (clinical SLEDAI-2K without serology = 0) with patients whit at least one active clinical manifestation at the time of enrollment (pooling different clinical manifestations together for the low number of subjects). This analysis showed that the presence of clinical activity does not influence the previous results (Supplementary Table 5).

Another limitation consists in the fact that the included patients presented heterogeneity in disease activity and treatment so being a potential bias of this study.

Taking these aspects into consideration, we have evaluated the correlation between the main clinical variables and the activity of P2X7R through a direct measure of its mechanism of action (represented by BzATP stimulated Δ[Ca^2+^]_i_-Fura-2/AM) and no significant correlations with disease duration, SLEDAI-2K and ongoing therapy were detected. In particular, in our series, 79% of patients were treated with hydroxychloroquine, a cornerstone in the SLE therapy, with an inhibitory action on TLR (Torigoe et al., 2018). Compared to the steroid, hydroxychloroquine accumulates in the body over a long period exerting a prolonged effect. To evaluate if this drug could have played a role in the reduction of the activity of P2X7R, we carried out a test on few samples in which PMBCs were pre-treated with 200 μM chloroquine and subsequently stimulated with BzATP to evaluate the Δ[Ca^2+^]_i_. The results obtained, showed that in the same subject, the chloroquine pre-treatment determined an increased Δ[Ca^2+^]_i_ suggesting that the reduced activity of P2X7R in SLE patients would not be due to the pharmacological action of this drug. Furthermore, despite the ongoing treatment and the absence of high disease activity, an increase of inflammatory cytokine IL-6 was found in patients both in supernatants of stimulated monocytes and plasma. The limited number of patients (especially those with serositis), and the wide variability among patients in cytokine expression, did not allow in some cases to reach statistical significance.

## Conclusion

Compared to auto-inflammatory diseases, in which inflammasome has the main pathogenetic role, SLE is a complex condition where alterations of adaptive and innate immunity coexist. During different phases of the disease, multiple pathways can influence each other and change their activity. Further studies should enroll patients at disease onset and naïve from therapy, to evaluate if P2X7R represents a bridging role between environmental risk factors and autoimmunity development in the early stages of the disease, especially in those patients where a trigger, for example infectious, is recognizable. In this case, P2X7R might be overexpressed during the initial phase of illness and downregulated once the autoimmune process becomes established. In this phase of the disease, other pathways and other cytokines, such as IL-6, could be prevalent and should be considered as important therapeutic targets in the treatment of SLE, especially in presence of clinical manifestations with a more “inflammatory” track as serositis.

## Ethics Statement

All subjects provided written informed consent. The study was approved by the local ethics committee and conducted in accordance with the amended Helsinki Declaration.

## Author Contributions

FF and AB formulated the concept and designed the manuscript. FF, ALG, and MP conducted the experiments. FF and ALG contributed to statistical analysis and wrote the manuscript. FF, ALG, MEP, MG, FD, and AB revised the manuscript critically, approved the final manuscript, and agreed to be accountable for all aspects of the manuscript.

## Conflict of Interest Statement

FD is a member of the Scientific Advisory Board of Biosceptre Ltd., a United Kingdom-based company involved in the development of P2X7-targeted antibodies, and has an ongoing collaboration with Ablynx, a Belgian company involved in the development of P2X7R-targeted nanobodies. The remaining authors declare that the research was conducted in the absence of any commercial or financial relationships that could be construed as a potential conflict of interest.
