# Supplementary material for: P2X7 Receptor Expression in Patients With Serositis Related to Systemic Lupus Erythematosus
Source: Front Pharmacol. 2019 Apr 29;10:435. doi: 10.3389/fphar.2019.00435 (PMC6501575; doi:10.3389/fphar.2019.00435)

Supplementary Tables and Figures

Supplementary Table 1: comparison between patients with (SLE-C) or without (SLE-NC) cutaneous lupus erythematosus (both chronic, subacute or acute lesions).

Supplementary Table 2: comparison between patients with (SLE-GMN) or without (SLE-NGMN) lupus nephritis

Supplementary Table 3: comparison between patients with (SLE-A) or without (SLE-NA) lupus arthritis

Supplementary Table 4: comparison between patients with (SLE-Neuro) or without (SLE-NNeuro) neuropsychiatric involvement

Supplementary Table 5: comparison between patients with active (SLE-Active) or past clinical manifestation (SLE-Past)

Supplementary Table 6: correlation between IL6 and CRP levels

Supplementary Figure 1: Representation of IL-1β and IL-6 plasma levels in SLE patients (SLE) compared to healthy controls (HC) and patients with serositis, without SLE. (A) IL-1β plasma levels were not statistically different between HC and SLE (SLE vs HC = 6.24±0.61 vs 5.42±0.38, p=0.384) and between SLE and patients with history of serositis, without SLE (SLE vs serositis = 6.24±0.61 vs 9.42±2.09, p=0.064). On the contrary, patients with serositis presented higher IL-1β plasma levels than HC (serositis vs HC = 9.42±2.09 vs 5.42±0.38, p=0.014). Unpaired t-test. (B) Subanalysis of SLE patients group, showed that IL-1β plasma levels were not statistically different between patients with history of serositis, without SLE and SLE-NS (serositis vs SLE-NS= 9.42±2.09 vs 6.39±1.13, p=0.201). On the contrary patients with serositis presented higher IL-1β plasma levels than SLE-S (serositis vs SLE-S = 9.42±2.09 vs 6.07±0.38, p=0.032). Unpaired t-test. (C) IL-6 plasma levels were not statistically different between HC and patients with history of serositis without SLE (serositis vs HC= 7.12± 4.12 vs 1.58±0.67, p= 0.067). On the contrary patients with serositis presented higher IL-6 plasma levels than SLE (serositis vs SLE= 7.12± 4.12 vs 3.09±0.57, p=0.024) were significantly higher in patients with serosits compared both to HC and SLE patients. Unpaired t-test. (D) Subanalysis of SLE patients group, showed that IL-6 plasma levels were not statistically different between patients with history of serositis without SLE and SLE-S (serositis vs SLE-S=

7.12± 4.12 vs 4.8±0.97, p=0.358). On the contrary patients with serositis presented higher IL-6 plasma levels than SLE-NS (serositis vs SLE-NS = 7.12± 4.12 vs 2.02±0.37, p=0.013). Unpaired t-test. *p < 0.05, **p < 0.01

Supplementary Figure 2. Representation of IL-1β levels in macrophages supernatants from SLE patients, healthy controls (HC) and patients with serositis without SLE. (A) IL-1β released from unstimulated macrophages maintained for 5 h in 10% FBS supplemented RPMI was not significantly different between HC, SLE patients and serositis patients (SLE-NS vs serositis= 16.63±5 vs 10.11±2.36, p=0.66; SLE-S vs serositis= 14.82±3.76 vs 10.11±2.36, p= 0.593; HC vs serositis=18.05±7.41 vs 10.11±2.36, p= 0.564). Unpaired t-test. (B) IL-1β released from macrophages stimulated for 4 h with 1 μg/ml LPS in 10% FBS supplemented RPMI was not significantly different between all groups of subjects and patients (SLE-NS vs serositis= 48.11±14.53 vs 48.75±17.33, p=0.99; SLE-S vs serositis= 55.38±13.47 vs 48.75±17.33, p= 0.83; HC vs serositis= 62.28±15.09 vs 48.75±17.33, p=0.67). Unpaired t-test. (C) IL-1β released from macrophages stimulated for 4 h with 1 μg/ml LPS and for 1 h with 300 μM BzATP in 10% FBS supplemented RPMI was not significantly different between HC and all the groups of patients (SLE-NS vs HC= 1237±70.39 vs 1246±58.61, p=0.936; SLE-S vs HC= 988.7±103.6 vs 1246±58.61, p= 0.072; serositis vs HC= 1402±104.4 vs 1246±58.61 p= 0.82; SLE-NS vs serositis= 1237±70.39 vs 1402±104.4, p=0.326). IL-1β release was on the contrary significantly lower in SLE-S compared to both SLE-NS (p=0.048) and serositis (p= 0.045). Unpaired t-test. (D) IL-1β released from macrophages stimulated for 1 h with 300 μM BzATP in 10% FBS supplemented RPMI was significantly lower in SLE patients respect to both HC and patients with serositis without SLE (SLE-NS vs serositis= 63.91±18.94 vs 318.7±125.8, p=0.0008; SLE-S vs serositis= 59.29±24.02 vs 318.7±125.8, p=0.047). No significant difference was found between HC and serositis patients (p=0.303). Unpaired t-test. Data are means ±SD. Only significant differences are shown. *p < 0.05, **p < 0.01, *** p < 0.005.

Supplementary Figure 3: representation of IL-6 levels in macrophages supernatants from SLE patients, healthy controls (HC) and patients with serositis without SLE. (A) IL-6 released from unstimulated macrophages maintained for 5 h in 10% FBS supplemented RPMI was not significantly different between HC and SLE patients vs serositis without SLE (SLE-NS vs serositis= 329.0±83.74 vs 14.37±1.71, p=0.21; SLE-S vs serositis= 29.18±5.66 vs 14.37±1.71, p= 0.18; HC vs serositis =11.83±0.51vs 14.37±1.71, p= 0.55). Unpaired t-test. (B) IL-6 released from macrophages stimulated for 4 h with 1 μg/ml LPS in 10% FBS-supplemented RPMI was not significantly different between HC and SLE-S patients vs serositis (SLE-S vs serositis= 728.8±101.2vs 811.1±110.73, p= 0.12; HC

vs serositis= 339.9±41.37vs 811.1±110.73, p=0.63). On the contrary IL-6 released was significantly lower in patients with serositis respect to SLE-NS (SLE-NS vs serositis= 1172±74.25 vs 811.1±110.73, p=0.0002. Unpaired t-test. (C) IL-6 released from macrophages stimulated for 4 h with 1 μg/ml LPS and for 1 h with 500 μM BzATP in 10% FBS-supplemented RPMI was not different between SLE patients vs serositis (SLE-NS vs serositis= 1159±83.87 vs 858.61±133.93, p=0.10; SLE-S vs serositis= 719.7±112.6 vs 858.61±133.93, p= 0.56) and was significantly lower in HC compared to serositis (HC vs serositis= 330.7±43.46vs 858.61±133.93, p= 0.0001). Unpaired t-test. (D) IL-6 released from macrophages stimulated for 1 h with 500 μM BzATP in 10% FBS-supplemented RPMI was not significantly different between HC and SLE patients vs serositis without SLE (SLE-NS vs serositis= 328±92.1 vs 75.32±58.52, p=0.08; SLE-S vs serositis= 43.95±9.49 vs 75.32±58.52, p= 0.75; HC vs serositis =13.38±0.76vs 75.32±58.52, p= 0.07). Unpaired t-test. Data are means ±SE. Only significant differences are shown. *p < 0.05, **p < 0.01, *** p < 0.005, ****p < 0.001.

Supplementary Figure 4: evaluation of Calcium influx with Fura2 in PBMCs after BzATP stimulation

The increase of [Ca2+]i (Δ[Ca2+]i) following stimulation with 500 μM BzATP was significantly higher in PBMCs from patients with serositis without SLE respect to HC (serositis vs HC= 191.8±38 vs 105.5±12.75; p=0.008) and SLE patients both SLE-S (serositis vs SLE-S= 191.8±38 vs 58.25±10.28;p=0.0016) and SLE-NS (serositis vs SLE-NS=191.8±38 vs 67.69±9.23; p=0.0005) *p < 0.05, **p < 0.01, ***p < 0.005.

Supplementary Table 1

	SLE-C (37;77%)	SLE-NC (11;23%)	p
**IL-1β plasma levels pg/ml; mean ±SD**	2.36±0.21	2.65±0.68	0.52
**IL-6 plasma levels pg/ml; mean ±SD**	1.38± 0.70	3.23±1.55	**0.03**
**IL-1β levels in macrophages supernatants pg/ml; mean ±SD**			
RPMI	97.78±139.26	35.36± 29.12	0.26
LPS	160.71±176.69	125.34±148.77	0.58
LPS + BzATP	2313.05±648.47	2528.43±751.38	0.42
BZATP	109.84±137.59	123.10±162.60	0.82
**IL-6 levels in macrophages supernatants pg/ml; mean ±SD**			
RPMI	667.29±874.65	647.88±833.80	0.95
LPS	2334.89±760.98	2352.66±755.54	0.95
LPS + BzATP	2456.65±937.93	2169.47± 726.70	0.40
BZATP	739.89±1004.26	565.06±875.74	0.65
**ΔCa^{2+} (Fura2) nM ±SD**	65.4± 16.8	69.12±41.59	0.85
**RT-PCR P2X7R mean ±SD**	0.85±0.44	1.19±0.40	0.28
**RT-PCR NLRP3 mean ±SD**	3.05±1.99	4.46±2.32	0.35

t-test and Mann Whitney test

Supplementary Table 2

	SLE-GMN (11; 34.4%)	SLE-NGMN (21;65.6%)	p
**IL-1β plasma levels pg/ml; mean ±SD**	2.4±0.12	2.5±0.2	0.63
**IL-6 plasma levels pg/ml; mean ±SD**	1.63± 0.77	1.85±1.31	0.84
**IL-1β levels in macrophages supernatants pg/ml; mean ±SD**			
RPMI	27.0±10.5	58.8±91.0	0.58
LPS	63.5±17.8	155.6±165.2	0.55
LPS + BzATP	645.3±189.2	2270.1±760.1	**0.003**
BZATP	155.8±116.8	109.6±141.1	0.21
**IL-6 levels in macrophages supernatants pg/ml; mean ±SD**			
RPMI	961.93±1091.95	419.16±455.35	0.11
LPS	2317.68±923.0	2363.64±601.15	0.88
LPS + BzATP	2399.69±916.57	2255.25±801.48	0.67
BZATP	913.28± 1166.03	435.81±647.58	0.22
**ΔCa^{2+} (Fura2) nM ±SD**	56.25± 55.09	72.78± 20.69	0.43
**RT-PCR P2X7R mean ±SD**	1.06±0.36	0.9±0.48	0.61
**RT-PCR NLRP3 mean ±SD**	3.86±2.31	3.31±2.14	0.72

t-test and Mann Whitney test

Supplementary Table 3

	SLE-A (31;64,6%)	SLE-NA (17; 35,4%)	p
**IL-1β plasma levels pg/ml; mean ±SD**	2.4±0.12	4±0.2	0.63
**IL-6 plasma levels pg/ml; mean ±SD**	2.16± 1.31	1.34±0.93	0.32
**IL-1β levels in macrophages supernatants pg/ml; mean ±SD**			
RPMI	86.8±132.20	48.74±63.23	0.51
LPS	149.89±180.28	135.19±129.92	0.83
LPS + BzATP	2468.07±586.91	2296.88±908.7	0.55
BZATP	113.54±132.91	121.59±181.61	0.89
**IL-6 levels in macrophages supernatants pg/ml; mean ±SD**			
RPMI	518.59±804.75	905.77±884.03	0.27
LPS	2432.41±822.02	2345.22±621.94	0.99
LPS + BzATP	2273.69±863.72	2400.77±836.33	0.72
BZATP	482.82±839.27	963.81±1027.74	0.21
**ΔCa^{2+} (Fura2) nM ±SD**	70.75± 36.05	62.8± 31.6	0.69
**RT-PCR P2X7R mean ±SD**	0.74±0.35	1.43±0.04	**0.01**
**RT-PCR NLRP3 mean ±SD**	2.99±1.86	4.59±2.49	0.28

t-test and Mann Whitney test

Supplementary Table 4

	SLE-Neuro (9;18;8%)	SLE-NNeuro (39; 81,2%)	p
**IL-1β plasma levels pg/ml; mean ±SD**	3.15±1.2	2.45±0.21	0.50
**IL-6 plasma levels pg/ml; mean ±SD**	2.6± 1.6	1.4± 0.76	0.15
**IL-1β levels in macrophages supernatants pg/ml; mean ±SD**			
RPMI	18.4±2.26	81.41±119.52	0.47
LPS	74.16±103.25	157.31±169.67	0.35
LPS + BzATP	1720.36±833.53	2528.49±613.68	**0.03**
BZATP	113.88±173.88	116.63±146.75	0.97
**IL-6 levels in macrophages supernatants pg/ml; mean ±SD**			
RPMI	257.88±357.54	734.18±855.55	0.31
LPS	2278.5±766.94	2355.78±756.49	0.85
LPS + BzATP	1910.2±460.23	2396.63± 878.92	0.29
BZATP	214.97±301.32	739.97±978.52	0.30
**ΔCa^{2+} (Fura2) nM ±SD**	87± 20.5	61.9± 34.95	0.26
**RT-PCR P2X7R mean ±SD**	1.03±0.57	0.85±0.38	0.56
**RT-PCR NLRP3 mean ±SD**	3.8±4.24	3.28±1.61	0.73

t-test and Mann Whitney test

Supplementary Table 5

	SLE-Active (16;33.3%)	SLE-Past (32; 66.7%)	p
**IL-1β plasma levels pg/ml; mean ±SD**	2.7±0.7	2.32±0.2	0.31
**IL-6 plasma levels pg/ml; mean ±SD**	2.1± 2.1	3.2± 2.1	0.39
**IL-1β levels in macrophages supernatants pg/ml; mean ±SD**			
RPMI	33.1±28.9	67.4±192.9	0.36
LPS	147.9±202.4	151.6± 150.7	0.94
LPS + BzATP	2256.3± 615.3	2227.6± 826.7	0.93
BZATP	63.7± 84.7	129.2± 156.5	0.18
**IL-6 levels in macrophages supernatants pg/ml; mean ±SD**			
RPMI	324.8± 405.7	489.1± 821.4	0.53
LPS	1866.9± 536.7	2087.6± 951.8	0.47
LPS + BzATP	1610.9± 551.4	2157.2±1011.9	0.10
BZATP	227.0± 304.7	539.5±894.8	0.26
**ΔCa^{2+} (Fura2) nM ±SD**	67.8± 38.7	64.6± 29.5	0.85
**RT-PCR P2X7R mean ±SD**	0.49±0.2	0.97±0.4	0.054

t-test and Mann Whitney test

Supplementary Table 6

	IL-6				
	Plasma	RPMI	LPS	LPS- BzATP	BZATP
**CRP**	-0.07; p=0.79	0.05; p=0.75	0.61; p=0.06	0.72; p=0.15	0.15; p=0.34

Spearmann correlation

Supplementary Figure 1

(A)

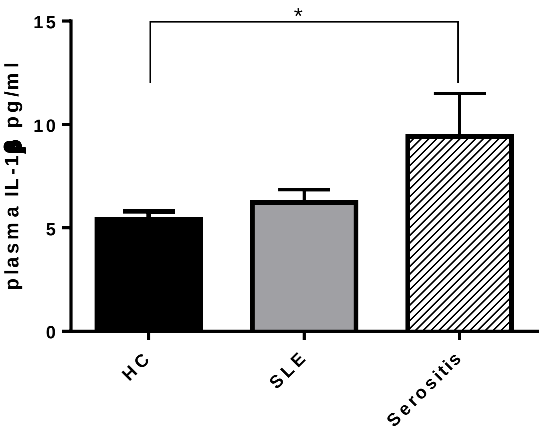

(B)

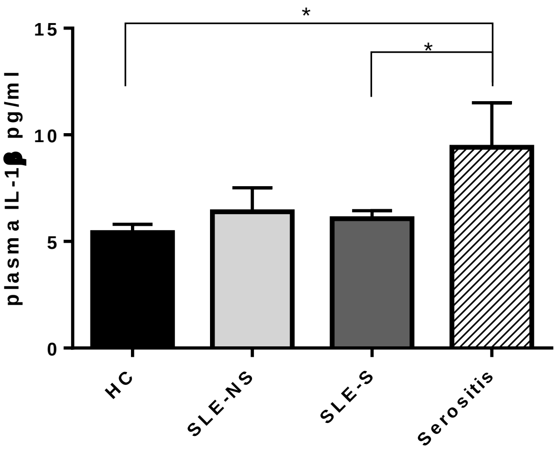

(C)

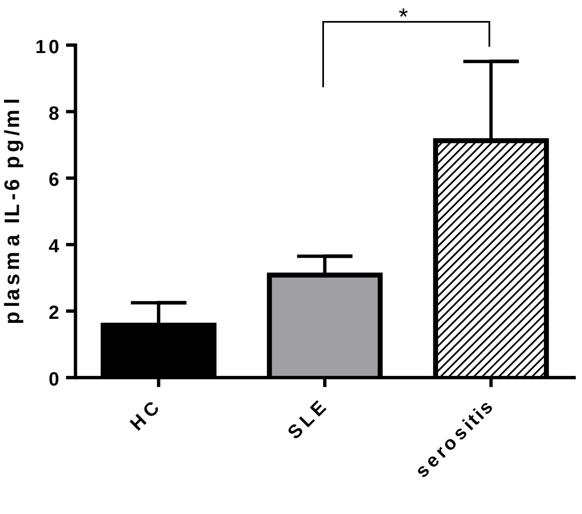

(D)

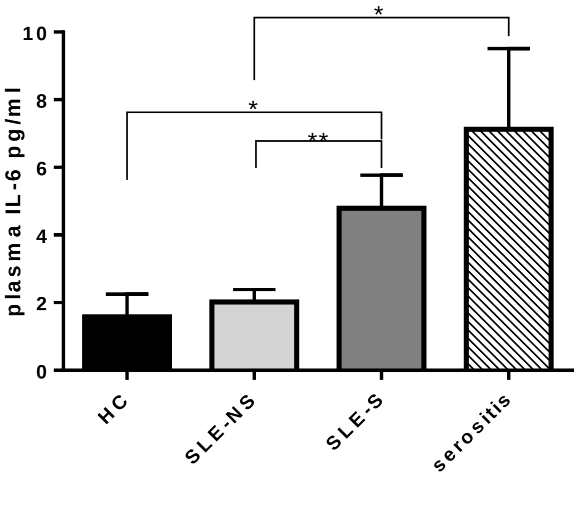

Supplementary Figure 2

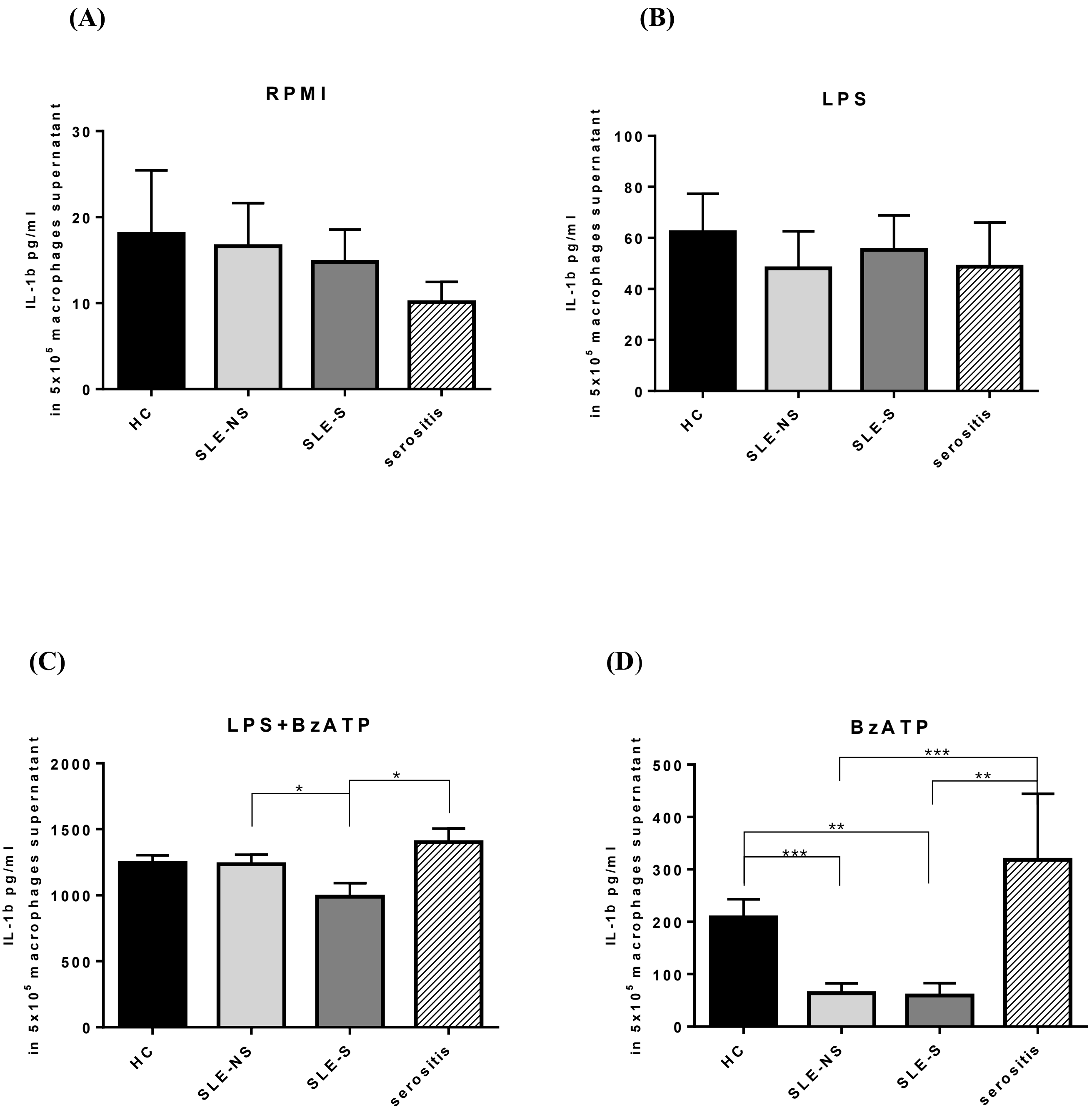

Supplementary Figure 3

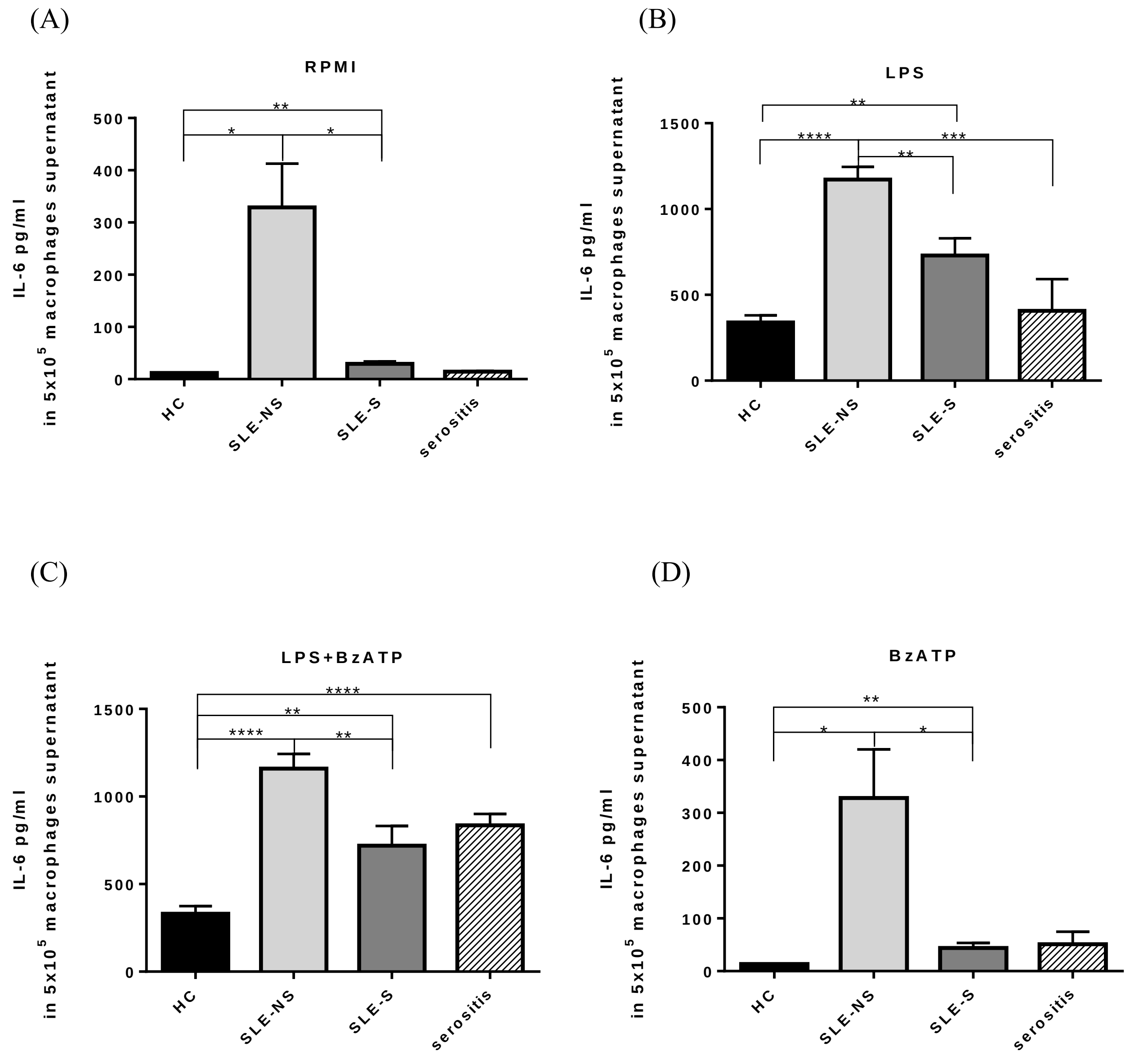

Supplementary Figure 4

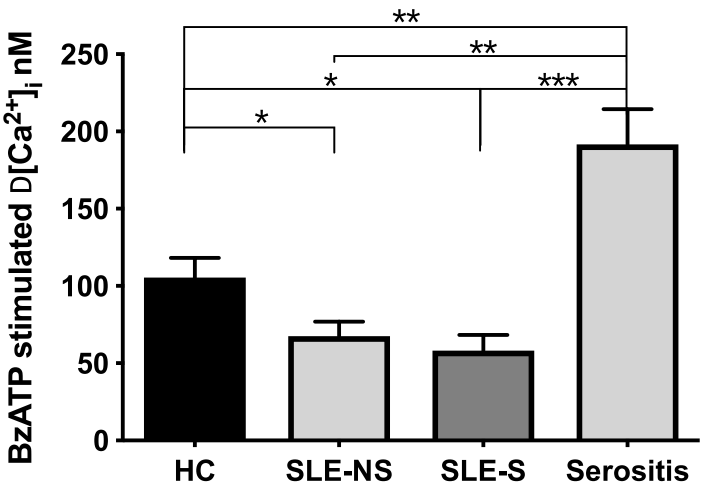

Supplement: Supplementary file 1 [file Data_Sheet_1.pdf]
